# Orbital B-cell Lymphoma Masquerading As Dry Eye Disease

**DOI:** 10.7759/cureus.37228

**Published:** 2023-04-06

**Authors:** Bharadwaj Adithya-Sateesh, Nicole Gousy, Rao Sateesh S Thimmanayakanahalli, Sang Tran, Rediet Tefera Atalay, Miriam B Michael

**Affiliations:** 1 Internal Medicine, American University of Antigua, St. John's, ATG; 2 Medicine, American University of Antigua, New York, USA; 3 General Medicine, Dr. Joseph Polyclinic, Dubai, ARE; 4 Ophthalmology, University of Maryland, Baltimore, USA; 5 Internal Medicine, Howard University Hospital, Washington DC, USA; 6 Internal Medicine, Howard University, Washington DC, USA; 7 Internal Medicine, University of Maryland, Baltimore, USA

**Keywords:** lymphoma, lymphoma of the eye, dry-eye, orbital proptosis, primary orbital lymphoma

## Abstract

Orbital lymphoma is a rare tumor with an incidence of 2.02 per million per year. It can occur as a part of systemic lymphoma or spontaneously arise primarily in orbit. The most commonly reported malignant lymphoma is non-Hodgkin’s lymphoma. The typical clinical finding includes exophthalmos, swelling, and limited eye movement. Our patient presented with dryness and irritation of the eye without signs of proptosis until months later. The definitive diagnosis of orbital lymphoma requires a biopsy. Still, imaging studies such as MRI and CT scans play a critical role in distinguishing benign from malignant lesions and invasion of local tissues. The treatment of orbital lymphoma is multidisciplinary, involving surgical resection, radiotherapy, and chemotherapy depending on the histological type of tumor and the presence of metastasis.

## Introduction

Orbital masses are rare clinical entities typically caused by infectious, inflammatory, and neoplastic etiologies. They can be either primary lesions stemming from the orbit itself, including the lacrimal gland, or secondary due to lesions involving the orbit by contiguity [1]. Adjacent structures such as the paranasal sinus, conjunctiva, eye, eyelid, lacrimal sac, face, anterior and middle cranial fossae, orbital bone, nasopharynx, palate, and parotid gland are commonly involved and, along with metastatic tumors, account for most secondary etiologies [1]. Orbital tumors are usually present in patients above 60 years old. The key symptom of an orbital tumor is proptosis, with other signs including eyelid swelling, edema, ptosis, conjunctival erythema, diplopia, and deficits in eye movement [1]. The most common orbital malignancy in the elderly population is a lymphoproliferative disease with a prevalence of 25-36% in the adult population [1]. Malignant lymphoma accounts for about 80% of orbital lymphoproliferative tumors and 24% of all space-occupying tumors in adults over 60 years of age [2].

Lymphoproliferative lesions may range from benign lesions such as reactive lymphoid hyperplasia or malignant lesions including lymphomas. Histopathology is required to differentiate benign lesions from malignant and other mimics such as orbital pseudotumors [2]. Benign lesions demonstrate well-differentiated lymphocytes with some pleomorphism, and malignant lymphomas have atypical cells with nuclear membrane abnormalities. Malignant lesions are usually B-cell in origin and require immunohistochemistry for exact diagnosis [2]. Extranodal marginal zone lymphoma (EMZL) is the most common subtype of B-cell originating orbital lymphomas, however, other common orbital lymphomas include diffuse large B-cell lymphoma (DLBCL) (23%), follicular lymphoma (FL) (9%), and mantle cell lymphoma (MCL) (5%) [3]. Gender does seem to play a role in the subtype of lymphoma, with EMZL and FL more common in females, MCL more common in males, and DLBCL having an even distribution [3]. The subtype of the lymphoma and the grade of the lesions are the best indicators of prognosis, with EMZL and FLs showing excellent prognosis and the higher grade lymphomas such as DLBCL showing poor prognosis [3]. Radiotherapy is the treatment of choice for low-grade lymphomas, whereas higher-grade lesions require chemotherapy with or without adjunct radiation [3]. EMZL and FLs have a relatively good prognosis, with high-grade lymphomas such as DLBCL or mantle lymphomas having a relatively poor prognosis. An exception is a potential transformation of EMZL to DLBCL, leading to a poorer prognosis [3].

## Case presentation

**Figure 1:**
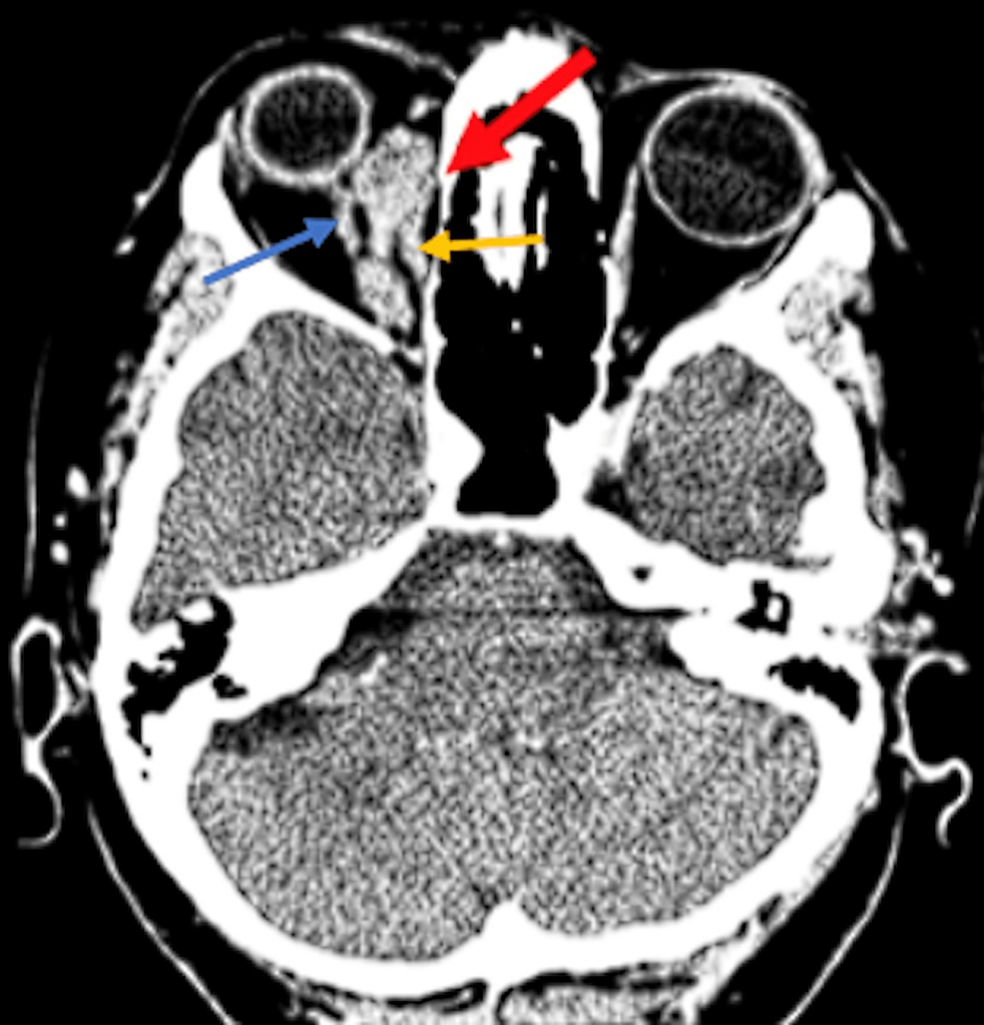
Axial CT of the patient during admission. Of note is the lobulated enhancing soft tissue mass lesion (red arrows) in the superomedial aspect of the right orbit with involvement of the right optic nerve (blue arrow) and the medial rectus muscle (yellow arrow) leading to mild proptosis. CT: computed tomography

**Figure 2:**
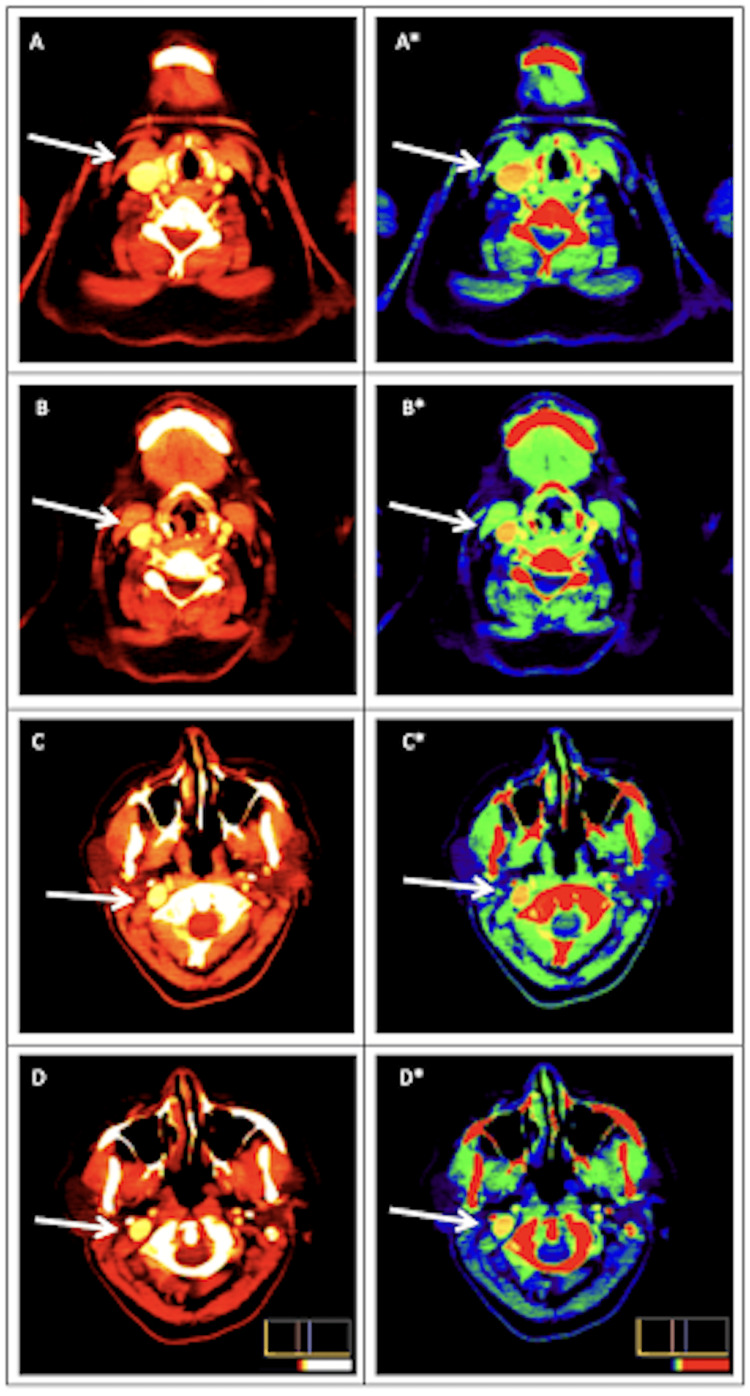
PET scan images reflecting increased uptake in the right anterior cervical chain (arrows). *Rainbow Scale PET: positron emission tomography

A 54-year-old female with a past medical history of hypothyroidism and type 2 diabetes mellitus presented to the primary care clinic with complaints of dryness and irritation of her right eye. A basic eye exam performed (which included testing for abnormalities in her visual acuity, pupillary light reflexes, and extra-ocular movements) revealed no significant findings. The patient was sent home with recommendations for an orbital warm compress. Two months later, she complained of a bump on her right eyelid along with dryness and was referred to an ophthalmologist. On examination, her extraocular muscles were functioning normally, and there was no appreciable proptosis. The patient revisited her ophthalmologist four months after the initial presentation with similar complaints of dryness and irritation, this time with mild 4.4mm proptosis on examination. A computed tomography (CT) scan of the brain and orbit showed a lobulated enhancing soft tissue mass lesion in the superomedial aspect of the right orbit involving the intraorbital portion of the right optic nerve and the medial rectus muscle which was causing the mild proptosis (Figure 1). A magnetic resonance imaging (MRI) of the brain and orbit performed to further evaluate the lesion revealed a moderately enhancing intraconal right orbital superomedial space-occupying lesion (SOL) with infiltration of the extraocular muscles, causing mild proptosis and caudal displacement of the eyeball (Figure 2). There was a lateral extension and thickening seen up to the lacrimal gland, with compression of the distal optic nerve and stranding of retrobulbar fat. The patient denied any visual changes or headaches, and had no deficits in her extraocular muscles, despite there being the involvement of the medial rectus muscle on the CT scan.

The imaging studies were inconclusive in making a diagnosis and tissue samples were needed for further evaluation. She underwent an orbitotomy and initial histopathology showed signs of a lymphoproliferative disorder versus granulomatous process. The further immunohistochemical evaluation showed a lymphoid infiltrate mostly consisting of CD20+ atypical B cells which were small to medium-sized with follicular expansion. The cells expressed Bcl2 but were negative for CD3, CD5, CD10, CyclinD1, and Bcl6. The findings were conclusive of low-grade EMZL. A positron emission tomography (PET) scan was performed which showed focal tracer uptake in the right half of the hard palate. There was also tracer uptake in the right anterior cervical lymph nodes making this a stage II EMZL. A plan was made in concurrence with the surgical and oncology teams to treat the patient with 20 sessions of targeted radiotherapy over the course of four weeks. A repeat PET was performed two months later and a repeat MRI six months later showed complete resolution of the EMZL.

## Discussion

Orbital lymphomas are rare and occur at an estimated incidence rate of 2.02 per million person-years, with only two thousand cases reported over the last 24 years [1,3]. There are six major pathologic contributors to the development of orbital mass lesions: inflammation, infection, hemorrhage, neoplasms, metastasis, and congenital causes [1]. Characterization of the mass can be divided into a primary orbital mass or secondary orbital mass based on their cellular origin. Primary orbital masses (82-87% of cases) originate from either the orbit or the lacrimal gland. Secondary orbital masses (9-11% of cases) are defined as originating from one of the neighboring orbital structures, including the paranasal sinus, the conjunctiva, the eyelid, the lacrimal sac, the anterior or middle cranial fossa, the orbital bone, the nasopharynx, the palate, or parotid gland. The remaining 4-8% of causes for orbital masses include metastatic tumors, most likely metastasizing from breast cancer, prostate cancer, or melanoma [1,2]. In adults, vasculogenic lesions, such as cavernous hemangiomas, are the most common cause of all ocular mass lesions [2]. In patients over 60 years of age, more than 50% of primary orbital tumors are lymphoproliferative lesions [1-3].

The most commonly reported malignant orbital lymphoma is non-Hodgkin lymphoma (NHL), specifically the mucosa-associated lymphoid tissue (MALT) subtype of B-cell origin (97%), followed by a mixed T/NK-cell origin (64%), with only 3% of cases reported being of T-cell origin [3]. These lesions can be an indication of systemic lymphoma or can spontaneously arise primarily in orbit. However, a primary NHL of the orbit is an exceedingly rare presentation, making up approximately 8-10% of extranodal NHL, and in the case of our patient, an even smaller 1% of all NHL in general [4]. Many cases tend to have unilateral lesions in the extraconal anatomical location. The typical clinical findings of patients with orbital lymphomas include exophthalmos of the affected eye, swelling, limited eye motility, visual acuity changes, diplopia, eye displacement, and “B-symptoms.” B symptoms are a related group of systemic symptoms, including fever, unexplained weight loss, or night sweats [3,4]. However, in our patient, the first symptom experienced was the sensation of eye dryness and irritation without apparent signs of proptosis until months later. While lacrimal involvement can be seen with orbital lesions (51% of cases) as seen in this case, there have not been any reports of insidious eye dryness and irritation without exophthalmos as the initial presentation of NHL orbital lymphoma, which is how this patient presented [3].

The low-grade EMZL is associated with several chromosomal abnormalities such as trisomies 3, 7, 12, and 18, somatic deletions or mutations of the A20 gene, and several chromosomal translocations [3]. While our patient did not have any genetic analysis completed, several studies have indicated a strong association with these chromosomal abnormalities [3]. It is thought that through these translocations, several regulatory genes are inactivated. This leads to a significant upregulation of proteins activating nuclear factor κB (NFκB), a crucial transcription factor deeply involved in adaptive and innate immunological processes that work through the promotion of cell survival via activation of induction genes [3]. The A20 gene, which also plays an inhibitory role in NFκB, has also been indicated in the development of EMZL due to somatic inactivation of the gene [3].

There is an association of EMZL with autoimmune disorders and other sources of chronic antigenic stimulation [3]. Several studies have reported the development of EMZL in patients with rheumatoid arthritis, Sjogren's syndrome, and systemic lupus erythematosus. Infections with one of several infectious organisms have been associated with the development of EMZL, including *Helicobacter pylori* in gastric EMZL, *Borrelia burgdorferi* in skin EMZL, *Chlamydia psittaci* (*C. psittaci*) in orbital cell lymphoma, and hepatitis C virus in ocular EMZL [3,5-7]. Previous studies have shown the resolution of gastric EMZL with the use of antibiotics, leading to the use of doxycycline as a treatment option for ocular EMZL. There are reports of complete remission in 7-22% of patients treated for ocular EMZL with doxycycline alone [5,7]. This may only apply to areas where *C. psittaci* is prevalent. A recent study revealed that in those with ocular lymphomas, the prevalence of *C. psittaci* is approximately 50% in Italy and Korea; 33% in Austria; 5-15% in China, Germany, the United Kingdom, France, India, the Netherlands, and Cuba; less than 5% in the United States; and 0% in Japan and Kenya [5]. However, another study reported finding DNA from *C. psittaci* in 80% of cases of orbital lymphoma [7]. This treatment modality may not apply to patients in India, such as this patient, but should be considered for patients who live in endemic areas. Since this patient was not residing within any endemic areas, she was not screened for any of these infectious causes of EMZL.

When orbital lesions are suspected, it is crucial to obtain imaging using both diffusion-weighted and dynamic contrast-enhanced MRI to distinguish benign lesions, such as inflammation, from malignant lesions [1]. Additionally, CT is essential in determining areas of calcification within the lesion or any bony erosions or other local tissue invasions that can indicate malignancy. CT imaging is also useful in characterizing the lesion anatomically, which can aid in the diagnosing process and explain any symptoms the patient is experiencing [1].

There are several variations of how different tumors and lymphomas can morphologically appear on imaging, making the identification of any orbital lesion challenging. The differential diagnosis of thrombosed varix cannot be ruled out with CT imaging but by having the patient perform the Valsalva maneuver during MRI. This would cause thrombosed varix to change size but not a mass lesion. A biopsy is the best way to identify the lesion and the cellular origins of any orbital mass. Lymphomas are generally small, and an open biopsy is typically the standard procedural protocol, as fine-needle aspiration is usually insufficient. In addition to biopsy, immunohistochemical identification is the preferred way to classify an orbital NHL. Testing for antibodies against CD3, CD5, CD20, and CD79α to identify a lymphoma is either of B-cell or T-cell origin [3]. Additional testing to further categorize the lymphoma can also include identifying antibodies against BCL2, BCL6, CD10, CD23, CD30, Cyclin D-1, MUM-1, and κ and λ light chains [3,6]. A PET scan adds utility in checking for metastatic spread [1]. The addition of targeted sequencing of the sample’s cellular genome to identify specific mutations leading to alterations in these proteins is becoming a necessary procedure in diagnosis.

The treatment of orbital NHL is multidisciplinary and depends on the histological type of the tumor and potential metastasis. For symptomatic lesions or those not metastatic, as in our patient, either surgical excision alone or in conjunction with radiotherapy is appropriate [7]. Typically, radiotherapy is used to either tumor eradication or decrease the size of the tumor for surgical excision. Surgery is effective for those with low-grade and localized lymphomas, such as EMZL [3,7]. However, in metastatic or disseminated cases, chemotherapy using a CHOP protocol (a multiagent protocol utilizing a combination of cyclophosphamide, vincristine, and prednisolone) is effective with significant long-term reduction in mortality and control of the disease [7].

## Conclusions

Orbital masses are exceedingly rare and can be classified as either primary, stemming from the orbit, or secondary. A typical initial presentation of an orbital mass can include proptosis, eyelid swelling, visual changes, and deficits in extraocular eye movement. However, in this case, the initial symptom leading to diagnosis was dry eye disease alone without associated visual changes or extraocular eye movement weakness, which subsequently progressed to the development of mild right eye proptosis. This unusual presentation of unilateral dry eye disease as an indicator of a rare disease highlights the importance of investigation into orbital masses in those with a higher risk of developing lymphoproliferative lesions of the orbit, especially when unilateral dry eye or exophthalmos is present. This includes patients over 60 years of age, those with autoimmune diseases, or those who reside in an area where specific bacterial infections are common. Earlier diagnosis of these patients can expedite treatment and further improve the patient prognosis for orbital lymphoproliferative lesions.
